# Development of *Psilocybe* Mushroom Species Reference Material—Cultivation Parameters and Chemical Profiles

**DOI:** 10.1093/jaoacint/qsaf007

**Published:** 2025-02-17

**Authors:** Coleton Windsor, Anna E Kreynes, Jeff S Chilton, William A Chioffi, Christopher Niebergall, Kelsey Dodds

**Affiliations:** North American Reishi, Ltd, DBA Nammex, Box 1780, Gibsons, BC V0N 1V0, Canada; North American Reishi, Ltd, DBA Nammex, Box 1780, Gibsons, BC V0N 1V0, Canada; North American Reishi, Ltd, DBA Nammex, Box 1780, Gibsons, BC V0N 1V0, Canada; North American Reishi, Ltd, DBA Nammex, Box 1780, Gibsons, BC V0N 1V0, Canada; North American Reishi, Ltd, DBA Nammex, Box 1780, Gibsons, BC V0N 1V0, Canada; North American Reishi, Ltd, DBA Nammex, Box 1780, Gibsons, BC V0N 1V0, Canada

## Abstract

**Background:**

Psilocybin-containing mushrooms are gaining the attention of the scientific community due to the potential benefits offered by their psychoactive phytochemicals in the treatment of addiction and various mental health conditions. Although there are hundreds of different *Psilocybe* species, only a handful have been successfully cultivated under indoor controlled conditions and chemically analyzed.

**Objective:**

The goal of this publication is to describe Nammex’s ongoing effort to cultivate poorly studied *Psilocybe* mushroom species and analyze them by high-performance thin-layer chromatography (HPTLC) to identify and quantify important psychoactive compounds.

**Methods:**

Pure mycelium cultures of *Psilocybe* species were created from spore prints and tissue of mushrooms collected in the wild. From these mycelia, numerous cultivars were developed and then propagated on various substrates, based on nutritionally supplemented cellulosic materials. Using indoor growth chambers under strictly controlled conditions, mushrooms were produced and prepared for analysis.

**Results:**

Six *Psilocybe* species (*P. zapotecorum*, *P. natalensis*, *P. azurescens*, *P. subaeruginosa*, *P. cyanescens*, and *P. stuntzii*) were successfully cultivated indoors. Species identity was confirmed through analysis of anatomical and microscopic features, as well as by DNA sequencing. HPTLC was successfully used to quantify psilocybin and psilocin and to identify norbaeocystin, baeocystin, and aeruginascin. *P. zapotecorum* had the highest psilocybin content (1.89%), and *P. stuntzii* the lowest (0.45%). Preliminary data showed that psilocybin concentrations remained stable across three successive flushes of *P. stuntzii*. Storage of fresh mushrooms in a −20 °C freezer prior to freeze-drying drastically reduced psilocybin and increased psilocin levels.

**Conclusions:**

This study successfully demonstrated the cultivation and chemical profiling of multiple *Psilocybe* species under controlled conditions. The detailed HPTLC analysis revealed species-specific differences in psychoactive compound concentrations. Future research will incorporate advanced techniques, such as HPLC and mass spectrometry, to develop a more comprehensive chemical profile of these mushrooms.

**Highlights:**

This study successfully cultivated and chemically analyzed six *Psilocybe* species, revealing species-specific differences in psychoactive compound concentrations. Storage conditions, differences between stem and cap, and mushroom developmental stage influenced the composition of psychoactive compounds.

Mushrooms are a treasure trove of nutritional and medicinal compounds used for centuries to promote health and longevity and to treat a wide range of conditions and ailments. Scientists have demonstrated antimicrobial (1–7), anticancer (2–9), anti-inflammatory, antioxidant, antihypertensive, and antidiabetic properties (2–7), with some species displaying neuroprotective, neuroregenerative (7, 10), and psychoactive effects (11–13). Underpinning these diverse physiological effects is the chemical diversity of mushroom compounds. For example, ß-glucans and ergosterol are widely distributed across the kingdom Fungi and are readily detected and analyzed through several methods, while other compounds are unique to particular species and require specific analytical methodologies for their identification and quantification.

The amount of bioactive compounds produced by a wild mushroom species, whether psychoactive or not, is determined by the genetics of the specific strain and by the environmental conditions under which it is grown. As such, a cultivated variety, or “cultivar,” is a genetically differentiated mushroom “strain” characterized by its geographic area, which encompasses the native climatic conditions and the substrate on which it feeds. Since strains of a mushroom species can have different qualitative and quantitative chemical profiles, the development of a stable cultivar is highly desirable to produce material that will yield consistent bioactivity. A stable cultivar is characterized by consistent growth characteristics, chemical composition, bioactive compound levels across multiple cultivation cycles, and reliable yields. The collection of a fresh mushroom or spore print from different locations is the starting point for the laboratory culture work required for cultivation necessary for the development of a novel cultivar.

There are hundreds of psilocybin-containing mushroom species in the world but only a small number are actively consumed, and fewer yet have been studied in detail for their psychoactive compounds. Approximately 10 native species have been used regularly by indigenous groups in Mexico (14), four to six species in Europe and Asia (13), and in more recent times, six to eight species have been documented to be consumed routinely in North America (15). Currently, the National Center for Biotechnology Information (NCBI) contains reference genomes for 12 different *Psilocybe* species (*P. cubensis, P subviscida, P. semilanceata, P. maluti, P. mexicana, P. zapotecorum, P. azurescens, P. allenii, P. subaeruginosa, P. caeruleorhiza, P. cyanescens,* and *P. tampanensis*), but the study of the chemistry, biodiversity, and cultivation practices of these mushrooms are still in the early stages.

Nammex is licensed by Health Canada to grow psilocybin-containing mushroom species. Both common and experimental methods of indoor cultivation are employed to grow different strains using various substrate combinations as well as lighting, temperature, and cropping cycle modulations, with the goal of developing high-yielding, genetically stable cultivars. As stable cultivars are established, analyses are performed to identify bioactive compounds that occur in sufficient abundance for accurate quantification.

In addition to the development of cultivation methods, cultivar selection, and optimal methods of specimen storage, efforts at Nammex include identifying and establishing scientifically validated methods of analysis as well as evaluating active compound abundance and stability in the dried mushroom form. Nammex is currently using high-performance thin-layer chromatography (HPTLC) for the quantification of psilocybin and psilocin in psilocybin-producing species, to capture chemical differences between individual species, across flushes of the same species, different mushroom parts (stem, cap), and mushroom developmental stages.

Currently, numerous companies, both legal and illegal, are successfully growing the pasture-inhabiting *P. cubensis* mushroom, due to ease of cultivation on its natural substrate of cow manure and composted cellulosic material, as well as its warm temperature habitat and adaptability to a wide range of environmental conditions. In contrast, other *Psilocybe* species such as *P. cyanescens* or *P. zapotecorum* present unique challenges, including more specific substrate requirements, different temperature preferences, and lower adaptability to controlled environments, making their cultivation more difficult and requiring optimized, species-specific methods. The indoor cultivation methods that we used to grow *Psilocybe* mushrooms closely mimic those of *Agaricus bisporus,* a commercially grown edible mushroom that also fruits naturally in pastures, and the availability of explicit, detailed instructions that were published and available since 1983 (16). Due to the widespread availability of cultivated *P. cubensis*, researchers and healthcare practitioners have enjoyed access to a high-quality, well-characterized supply of this psychoactive mushroom. Considering this fact, Nammex has chosen to focus on cultivating psilocybin-containing mushroom species that are less well known and not available in sufficient quantities nor well characterized analytically. This will allow Nammex to provide researchers with standardized material for more detailed analysis of the complex array of compounds present in these species.

The work presented here documents initial stages of establishing the chemical profile and species variation of cultivated *Psilocybe* mushrooms as well as some of the techniques used for the generation of high-quality cultivars and their cultivation.

## Experimental

### Cultivation of Psilocybe Mushrooms

- *Cultivar development.*—*Psilocybe* mushroom cultivars were developed from wild-collected mushroom specimens and spore prints. Tissue and multispore mycelium cultures were grown in Petri plates, on a malt extract agar, containing 20 g malt syrup and 25 g agar per liter of water. Fast-growing mycelial sectors were selected from the original plates and placed in new Petri plates for further expansion and growth.
- *Substrate preparation and inoculation.—*Substrate combinations were formulated based on observed species preferences in their natural environment and materials commonly used in commercial mushroom cultivation. Primary materials were deciduous wood pellets (sawdust), alder wood chips, soy hulls, coco coir, straw, steer manure, rye grain, and corn cobs, all purchased at a local farm and garden store. All materials were screened or milled to approximately 10–20 mesh as needed to conform to a structural density that allowed sufficient aeration for unimpeded mycelial growth. Additional nutrients such as rice or wheat bran, corn molasses blend, soybean meal, and cottonseed meal were added to supplement the base materials and achieve specific carbon nitrogen ratios. Agricultural limestone (calcium carbonate) was added as necessary to reach a neutral state. All the materials were blended and soaked in water to an approximate 60% moisture content. The blended substrates were filled into standardized autoclavable 0.2 µm filter patch polypropylene bags (Unicorn Bags, 4 T) in 1 or 1.5 kilo amounts. The substrate-filled bags were steam-sterilized in an autoclave at 15 psi for 2 h, and then cooled to room temperature prior to inoculation.
  For each cultivar, healthy, abundant mycelial growth was sectioned from Petri plates and homogenized in sterile distilled water for 4–8 s in an Eberbach blender. The resulting liquid suspension was injected into each opened substrate bag at a volume of 50–100 mL, using a Wheaton self-refilling syringe. Each bag was closed, and the inoculated substrate was shaken to evenly distribute the liquid, after which the inoculated bags were shelved and incubated at 26 ± 2°C.
- *Incubation and growth conditions for fruiting body production.—*Approximately 14–21 days after inoculation, or once the mycelium has completely grown throughout the substrate, the bags were placed inside clear plastic storage bins, four to six bags per bin, with a standard bin size of 32 liters, 46 × 38 × 27 cm. At this time, the grow bags were opened and the top half of the bag removed to expose the surface of the fully colonized substrate. A 1–2 cm deep soil-like casing layer, consisting of sterilized, pH-neutral potting soil mix or similar soil-like mixture, was spread over the surface of the colonized substrate. After casing, the substrate bags continued to incubate until the mycelium reached the surface, before being transferred to the mushroom fruiting space shelves, where appropriate temperature, light, and humidity conditions are present.
  Mushrooms were propagated in one of two different enclosures, depending on temperature requirements. Cold temperature species were grown in Enclosure 1, a 200 square foot insulated shipping container with chrome-plated metal shelving, 30′′ wide by 20′ long. Each set of shelves had four tiers for a combined surface area of 400 square feet. Temperature control was provided by a 12-ton heat pump (Fujitsu) that supplied forced-air heating and cooling. Fresh air was regulated by a small, one square foot, filtered opening in the wall near the heat pump. Humidity inside the plastic grow bins was maintained at 90 ± 5% by watering and air flow adjustment. Enclosure 1 was kept at a regular temperature of 15 ± 2°C for cool-weather species adapted to northern climates. Monitoring of temperature and humidity was performed remotely by multiple wireless sensors (Aqara) in addition to four standard digital temperature/humidity instruments placed throughout the chamber. Lighting was supplied by a row of overhead T8 full-spectrum LEDs with a 6500K color temperature. Supplemental lighting was supplied by LED 6300K flexible rope lights (Shine Decor). Complete lighting in this enclosure was programmed to a 12-hour on/off schedule. For warm-temperature species, we employed Enclosure 2, a Conviron (G1000 Grow Chamber), with inner dimensions of 52″ H × 38″ W × 24″ D and with programmable controls for temperature, lighting, fresh air, and relative humidity. This Conviron was set at 20°C, 85% RH, and a 12-hour lighting schedule.
  The mushroom growing bins were monitored daily, and water was sprayed on the casing surfaces inside the bins to maintain the high humidity necessary to support the formation of primordia and provide water needed for mushroom growth and maturation. Growth stages were carefully observed and monitored. Mature mushrooms were harvested while the cap was still conical to convex by twisting them out of the casing layer or substrate. Any residual casing or substrate remaining on the stem was removed and discarded. Harvested mushrooms were freeze-dried in a freeze-dryer (Harvest Right HRFD LPH) and placed inside a sealed moisture-resistant plastic bag. The bags were stored at room temperature in the dark inside a safe.
- *Psilocybe species identification.—*Species identity was confirmed by analyzing anatomical features of different mushroom parts, through visual observation and microscopic analysis. For microscopic analysis, fruiting bodies were collected from mature specimens and caps were dissected under a dissecting scope (CELESTRON Labs) to obtain gill cross-sections. Sliced tissue was stained with methylene blue to improve contrast and mounted in water on glass microscope slides. Microscopic features were analyzed using OMAX M83E series LED Lab Trinocular Compound microscope using 1000× magnification and were photographed using OMAX 5.2MP microscope-mounted camera. ToupView X64 software was used to process photographs and evaluate dimensions of key morphological features (i.e., spores). Spore measurements were made in profile view, and dimensions were calculated as described in the literature (17–20), and the values reported as follows: (min. length outlier) 10 percentile–mean length–90 percentile (max length outlier) × (min. width outlier) 10 percentile–mean width–90 percentile (max width outlier), N = sample size, Q (quotient) value is the mean length divided by the mean width. Calculations for each species are listed below in micrometers (μm).
*P. azurescens*: (10.1)10.8–11.4–11.8(12.0) × (5.8)6.0–6.5–6.9(7.3), N = 9, Q = 1.75; *P. cyanescens*: (9.6)10.0–10.3–10.6 × (6.3)6.4–7.1–7.4(7.6), N = 12, Q = 1.45; *P. natalensis*: (8.8)9.0–9.5–9.9(10.1) × (5.5)5.6–5.8–6.0, N = 9, Q = 1.64; *P. stuntzii*: (6.6)7.2–8.0–9.1(9.6) × 4.7–5.6–6.6, N = 9, Q = 1.42; *P. subaeruginosa*: (10.0)10.5–11.1–11.9(13.7) × (5.7)5.9–6.4–6.5(7.8), N = 14, Q = 1.75; *P. zapotecorum*: (4.5)4.7–5.1–5.4(5.7) × (2.5)3.1–3.2–3.4(3.6), N = 18, Q = 1.59.
  To corroborate our examination of anatomical features and confirm the identity of the *Psilocybe* species used in this study, DNA testing was performed on all six of the cultivated species. For each species, a fresh mushroom was crushed into a Whatman^TM^ card (GE Healthcare, Cat No. WB1200065) and sent for DNA extraction and analysis by independent third-party laboratories. DNA identification of *P. azurescens, P. stuntzii, P. subaeruginosa, P. natalensis*, and *P. zapotecorum* was performed by LeafWorks (California, USA), while *P. cyanescens* was identified by Exact Scientific Services (Washington, USA).

### Chemical Analysis

- *HPTLC chemicals and reagents.—*Chemical reference standards were purchased from Cayman Chemical (Ann Arbor, MI, USA): psilocybin (CRM; Item No. 14041, Batch No. 0657546), psilocin (CRM; Batch No. 0667869), psilocybin, ≥98% (Item No. 9003134, Batch No. 0591153–1), baeocystin (Batch No. 0633877–12), norbaeocystin (Batch No. 0675226–8), aeruginascin (Batch No. 0622033–19). Additional standards were obtained from Sigma Aldrich: tryptamine (Batch No. BCCH6377), L-tryptophan (Batch No. BCCG9306).
  HPTLC solvents were obtained from Sigma Aldrich and include methanol ACS reagent, ≥99.8%, 1-butanol 99%, and acetic acid glacial, ACS reagent, ≥99.7%. Reagents used for preparation of derivatizing reagents: p-anisaldehyde, ≤100% (Sigma Aldrich) and sulfuric acid 98% (Sigma Aldrich).
- *Preparation of Psilocybe mushrooms.—*To prepare test samples for HPTLC fingerprinting, lyophilized mushrooms were ground finely with mortar and pestle, and 100 mg (± 1 mg) of the mushroom powder transferred into a 15 mL centrifuge tube. Subsequently, 4 mL of methanol and acetic acid (95:5, v/v) was added as the extraction solvent. The suspension was vortexed for 10 s, followed by sonication at room temperature for 15 min. The sample solutions were centrifuged at 1480 × *g* for 10 min and the supernatant was transferred into HPTLC vials. This procedure yielded a final extract concentration of 25 mg of dried mushroom mass per mL extraction solvent.
- *Preparation of HPTLC standards, mobile phase, and derivatizing reagents.—*Standard solutions of psilocybin, psilocin, baeocystin, norbaeocystin, aeruginascin, tryptamine, and L-tryptophan were prepared by dissolving the chemical reference standards described above (*HPTLC Chemicals and Reagents, a*) in methanol to appropriate concentrations.
  The mobile phase used for this study’s HPTLC analysis was a mixture of 1-butanol, acetic acid, and H_2_O in a ratio of 6.5:1.75:1.75 (v/v/v) adapted from an existing method (21).
  Anisaldehyde derivatization reagent was prepared by mixing 20 mL of acetic acid with 170 mL of ice-cold methanol, followed by 10 mL of sulfuric acid, and finally 1 mL of anisaldehyde.
- *Instrumentation, HPTLC parameters, and chromatographic conditions.—*The following HPTLC equipment and software from CAMAG (Muttenz, Switzerland) were used: Automatic TLC Sampler (ATS 4), Automatic Development Chamber (ADC 2), TLC Scanner 4, Plate Heater 3, TLC Visualizer 2, Immersion Device 3, and VisionCats 4.0 software. Silica gel 60F_254_ HPTLC Premium Purity 20 × 10 cm glass plates (Merck, Darmstadt, Germany).
  Aliquots were applied as narrow bands of 8.0 mm length at 8.0 mm from the lower edge of the plate. The bands of the outer lanes were applied 20 mm from the edge of the plate. The distance between tracks (center to center) was 11.0 mm. Application volumes were 10.0 µL. For identification testing, each chemical reference standard was applied at 2 µL.
  Development conditions were as follows: 30 s of pre-drying, a chamber temperature of 22 ± 5 °C, activation for 10 min to a relative humidity of 35% (±5%) (achieved using saturated MgCl_2_), 20 min of mobile phase saturation with pad, plate development to 70 mm, and 5 min of drying.

### Calibration and Quantitative Analysis of Psilocybin and Psilocin

**Figure 1.**
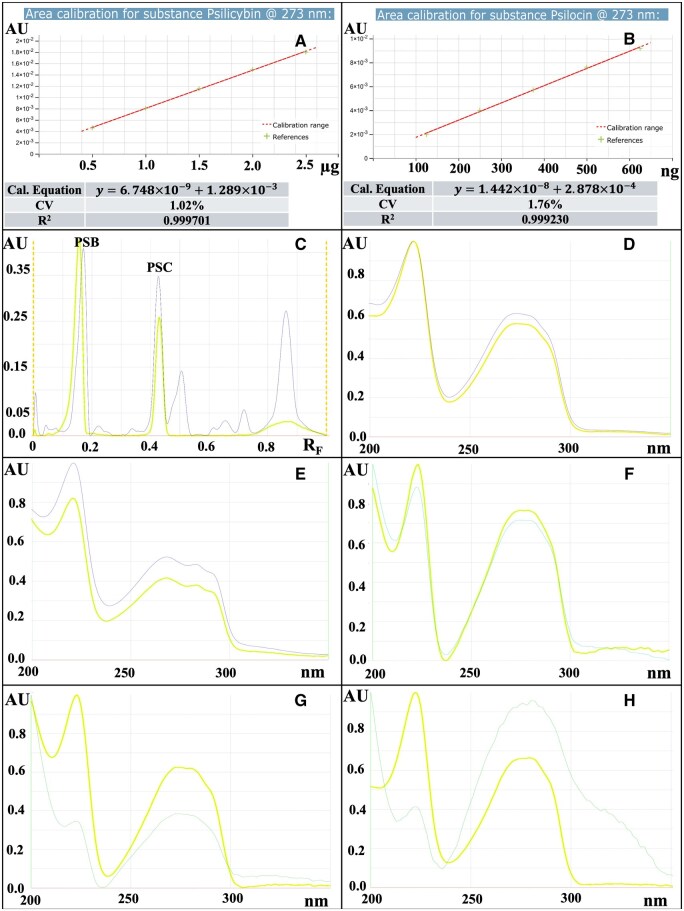
HPTLC calibration curves and statistical parameters for psilocybin and psilocin. Calibration curves for psilocybin (A) and psilocin (B), with coefficient of variation (CV), calibration curve equation, and correlation coefficient, R^2^. HPTLC-Densitometry absorbance chromatogram at 273 nm of *Psilocybe* mushroom extract, with psilocybin and psilocin bands highlighted in yellow (C). Absorbance spectra of chemical standards and corresponding bands extracted from psilocybin mushrooms under cultivation, at specific R_F_ values: psilocybin PSB (D), psilocin PSC (E), baeocystin (F), norbaeocystin (G), and aeruginascin (H).

- *Calibration curve preparation.—*Linear calibration curves for psilocybin and psilocin were prepared by plotting peak area versus concentration of chemical reference standard. Psilocybin calibration curves were established using four or five points ranging from 0.5 to 2.5 µg/band, while psilocin calibration curves used four to five points between 0.125 and 0.625 µg/band.
- *Quantification of psilocybin and psilocin.—*Quantification of psilocin and psilocybin was performed using densitometric scanning at 273 nm following plate development. While the primary absorbance at 222 nm was more intense, 273 nm was chosen to minimize potential interference from other compounds present in the matrix and to provide greater specificity for psilocybin and psilocin. Using VisionCats HPTLC software, peak areas were integrated and compared to the linear calibration curves to determine the concentrations of psilocybin and psilocin in the test solutions.
- *Data analysis procedures.—*Refer to Figure 1A and B for a summary of the linear regression evaluations of the psilocybin and psilocin calibration curves, including the curve equations, coefficients of variation (CV), and correlation coefficients (R^2^). The limits of detection (LOD) and limits of quantification (LOQ) were determined using the standard deviation of the response (σ) and the slope of the calibration curve (S), applying the formulas: LOD = 3.3σ/S and LOQ = 10σ/S. For psilocybin, the LOD and LOQ were calculated as 63.8 ng/band and 193.4 ng/band, respectively. For psilocin, the LOD and LOQ were 26.5 ng/band and 80.2 ng/band, respectively.
  Specificity was evaluated by comparing the R_F_ values of psilocybin and psilocin reference standards with corresponding bands in a *Psilocybe* mushroom test solution (Figure 1C). To confirm the identity of the compounds in the *Psilocybe* mushrooms, spectral analysis was performed by comparing the UV-Vis spectra of chemical reference standards for psilocybin, psilocin, aeruginascin, baeocystin, and norbaeocystin with the corresponding bands in the sample extracts (Figure 1D–H). In each case, the spectra of the compounds in the samples matched the standards, exhibiting identical absorption maxima.

### HPTLC Chemical Fingerprint Analysis

Following densitometric scanning for quantification, plates were immersed in the derivatizing reagent, with the Immersion Device 3 set to speed 5 and time 0. Immediately following the immersion, the plate was placed on the Plate Heater 3 at 100°C for 3 min. The derivatized plates were imaged under white light reflectance and 366 nm UV light to capture detailed chemical fingerprints of the mushroom test solutions.

These fingerprints were used to identify major compounds present in the *Psilocybe* mushrooms, including psilocybin, psilocin, norbaeocystin, baeocystin, aeruginascin, tryptamine, and tryptophan. For each species tested for HPTLC fingerprinting, between 2 and 12 mushrooms were used, depending on specimen size and availability.

## Results and Discussion

### Mushroom Cultivation

**Figure 2.**
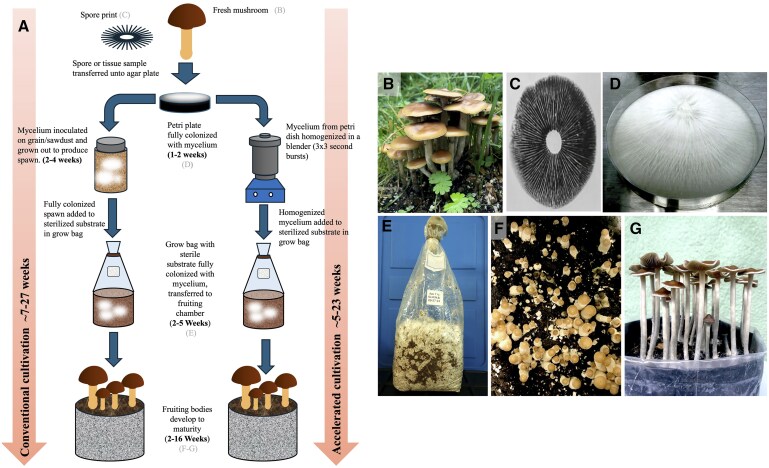
Stages of mushroom cultivation. Diagrammatic representation of mushroom cultivation stages (A) depicting conventional (left-hand side) and accelerated (right-hand side) protocols. Wild mushroom specimens (B) isolated from their natural environment were used to obtain a spore print (C), or tissue for culturing in the laboratory, under sterile conditions. Mycelium propagated from a multispore plate or tissue culture, growing on a Petri plate (D). Grow bag containing sterilized substrate, colonized with mycelium (E). Young mushrooms (button stage) developing on a cased substrate (G), and mature mushrooms ready to harvest (F), all grown indoors under controlled conditions.

Cultivating mushrooms from a spore print poses challenges due to the genetic diversity that arises from multispore germination, which can affect morphology and cultivation adaptability. Multispore germination on Petri plates often results in various mycelial sectors, some fertile and others not, necessitating multiple isolations to identify viable strains. Alternatively, tissue isolation from a fresh mushroom can be used to propagate mycelium, offering a higher probability of successful cultivation. However, even with this method, successful fruiting is not guaranteed as some species are inherently difficult to cultivate. Because of these challenges, it is important to optimize and streamline cultivation protocols, when possible, to save time. Typically, mycelium is transferred to sterilized grain or sawdust and grown for 2–4 weeks to create mushroom spawn, which serves as a seed for subsequent inoculation of the substrate. We successfully bypassed this step by homogenizing Petri plate–grown mycelium in sterile water and injecting it directly into the substrate, improving efficiency by eliminating the spawn production stage, thus shortening the cultivation timeline by 2–4 weeks. A comparative diagram of conventional cultivation and our accelerated protocol is depicted in Figure 2A, with corresponding images from different stages of cultivation displayed in Figure 2B–G, beginning with a wild specimen (Figure 2B), from which a spore print is obtained (Figure 2C). Mycelium culture (obtained from a germinated spore or tissue culture) is grown on a Petri plate until it is fully colonized (Figure 2D), before getting homogenized and inoculated into a grow bag containing sterile substrate, which is rapidly colonized by the mycelium (Figure 2E). Once fully colonized, the grow bag is cased and transferred to a chamber, with appropriate fruiting conditions for that species, for primordia initiation and eventual harvesting of mature cultivated mushrooms (Figure 2F–G).

**Figure 3.**
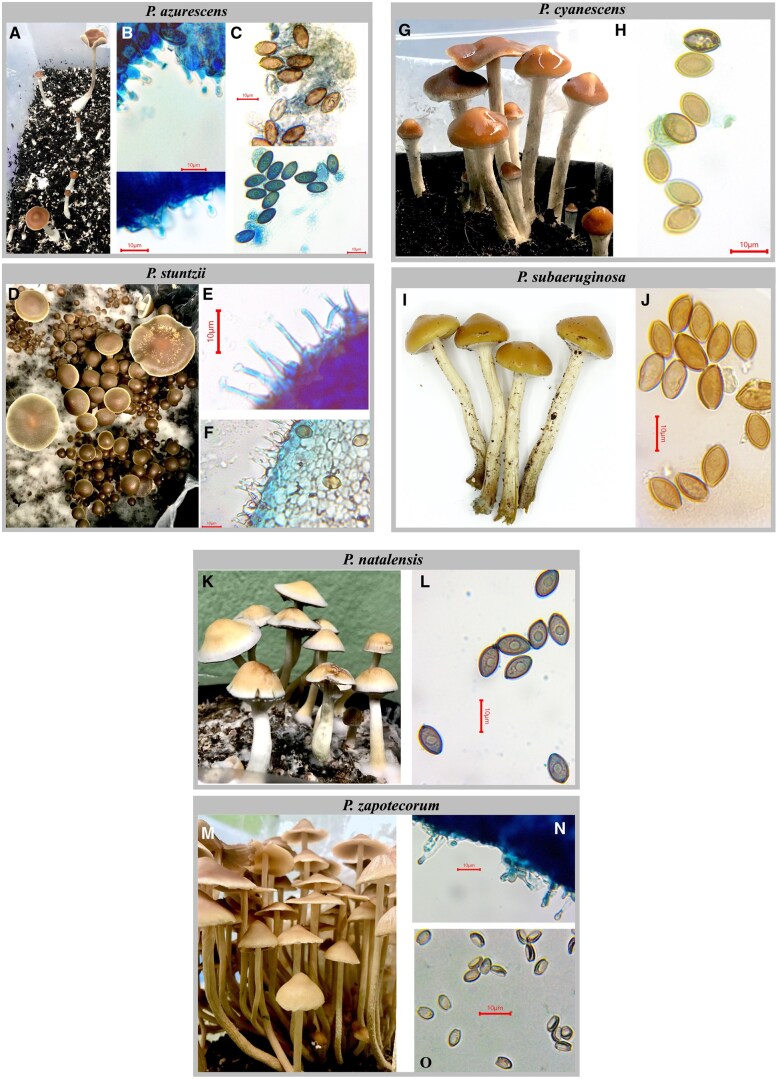
Mature mushrooms and microscopic features of six *Psilocybe* species under cultivation*. P. azurescens* (A), cheilocystidia (B), and basidiospores (C). *P. stuntzii* (D), cheilocystidia (E), and basidiospores (F). *P. cyanescens* (G) and basidiospores (H). *P. subaeruginosa* (I) and basidiospores (J). *P. natalensis* (K) and basidiospores (L). *P. zapotecorum* (M), cheilocystidia (N), and basidiospores (O). Microscopic images in panels B, C (lower) E, F, L, and N depict tissue stained with methylene blue.

There were notable differences in mycelial growth rates on Petri plates among different species, and in their ability to produce mushrooms. For instance, *P. baeocystis* (not featured in this paper) tissue culture was growing slowly in vitro and on substrates and has not yet yielded mushrooms, whereas a similar species, *P. stuntzii,* started from a spore print, grew quickly in vitro, colonized its substrate, and produced prolific flushes of morphologically perfect mushrooms (Figure 3D). Other prolific mushroom producers include *P. subaeruginosa* (Figure 3I), *P. natalensis* (Figure 3K), and *P. zapotecorum* (Figure 3M)*. P. azurescens* (Figure 3A) and *P. cyanescens* (Figure 3G) showed initial sporadic mushroom growth; however, *P. cyanescens* produced flushes of high-quality mushrooms after an extended period of time.

To date, Nammex has successfully cultivated indoors, under controlled conditions, the following species of *Psilocybe* mushrooms: *P. azurescens, P. cyanescens, P. natalensis, P. stuntzii, P. subaeruginosa*, and *P. zapotecorum* (Figure 3). We also have live mycelium cultures of eight other species in various stages of development. The successfully cultivated species seen in Figure 3 yielded sufficient material for chemical analysis, enabling identification and quantification of key chemical constituents.

### Species Identification

Morphological features of mature mushrooms, as well as microscopic features (including basidiospores and cheilocystidia), are described in Tables 1 and 2, respectively, and depicted photographically in Figure 3. Mature mushrooms of *P. azurescens* (Figure 3A), *P. stuntzii* (Figure 3D), *P. cyanescens* (Figure 3G), *P. subaeruginosa* (Figure 3I), *P. natalensis* (Figure 3K), and *P. zapotecorum* (Figure 3M) displayed morphological features characteristic of their species, as described in the literature (17–20). Images of cheilocystidia were obtained from *P. azurescens* (Figure 3B), *P. stuntzii* (Figure 3E, F), and *P. zapotecorum* (Figure 3N), and their distinct shapes appear as described in the literature (17). Spore appearance for all species corresponded to the descriptions in the literature (Table 2, and Figure 3C, F, H, J, L, O). Spore sizes of *P. stuntzii, P. cyanescens*, and *P. subaeruginosa* fell in the range of anticipated spore width and length for each species (17); however, spores of *P. azurescens*, *P. natalensis*, and *P. zapotecorum* were smaller than anticipated (Table 2). One possible explanation for this is that some of these spores are not fully mature, as mushrooms were frequently harvested before the cap was fully open. Additionally, the relatively small sample size of spores examined could have led to these results, and can be remedied as more material becomes available for examination. Despite this discrepancy, all other morphological features, as well as DNA identification, corroborated that the species under cultivation have been accurately identified.

**Table 1.** Macroscopic identifying features of *Psilocybe* mushrooms under cultivation^a^

	Macroscopic features			
Species	Pileus/cap	Lamellae/gills	Stipe/stem	Veil
*P. azurescens*	D: 3–10 cm^b^ Young cap conic, expanding to broadly convex/flat at maturity. Color: ochre-brown, caramel. Figure 3A	Attached. Color: cream to light brown Figure 3A	L: 9–20 cm^c^ W: 0.3–0.6 cm wide^d^ Color: white, with light browning near bottom at maturity Figure 3A	Absent Figure 3A
*P. stuntzii*	D: 1.5–5 cm Young cap obtusely conical, expanding to become umbonate, nearly flat at maturity. Color: dark chestnut, with light edges. Figure 3D	Narrowly attached with three tiers of intermediate gills.	L: 3–60 cm W: 0.2–0.4 cm Color: yellowish-brown Figure 3D	Partial veil thinly membranous. Leaves a ring on mature stipe.
*P. cyanescens*	D: 2–5 cm Young cap is conical, expanding to umbonate with wavy edge at maturity. Color: caramel Figure 3G	Broadly attached. Color: cinnamon-brown, darkening with age.	L: 8 cm W: 0.5 cm Color: white, bruising blue Figure 3G	Present when young, light cobweb appearance. Barely visible when mature. Figure 3G
*P. subaeruginosa*	D: 1–5 cm Young cap conic, expanding to convex/umbo at maturity Color: golden brown turning dark brown with age Figure 3I	Varying from broadly attached to narrowly attached to the stipe.	L: 5–12.5 cm W: 0.2–0.5 cm Slightly swollen at the base, fleshy white and bruising blue Figure 3I	Partial veil when young, leaving little to no trace on the mature stipe. Figure 3I
*P. natalensis*	D: 1.5–6 cm Young cap obtusely conical, expanding to convex at maturity. Color: yellow center with white edges. Figure 3K	Bluntly attached.	L: 4–12 cm W: 0.2–1 cm Silky white and bruising bluish-green Figure 3K	Partial veil present when young, scant to absent at maturity
*P. zapotecorum*	D: 1–3 cm H: 7–11 cm^e^ Variable form. Young cap conic/convex to subumbonate, sometimes expanding to papillate with age. Figure 3M	Broadly attached to notched, pale brown to purple with age Figure 3M	L: 4–20 cm W: 0.5–1.5 cm wide. White to gray or pale brown. Velvet texture near base Figure 3M	Absent

a Descriptions of freshly cultivated *Psilocybe* specimens are based on the vocabulary used in the literature (17, 19, 20).

b D = Diameter.

c L = Length.

d W = Width.

e H = Height.

**Table 2.** Microscopic features, spore size, and DNA identification of *Psilocybe* species under cultivation^a^

	Microscopic Features/DNA ID				
Species	Cheilocystidia	Spore shape	Spore size observed, µm	Spore size reported, µm	DNA ID
*P. azurescens*	Abundant, fusoid-ventricose (swollen/enlarged middle), tapers to a narrow short neck. Figure 3B	Ellipsoid Figure 3C	L: 10.8–11.8^b^^,d^ W: 6.0–6.8^c^^,d^ Figure 3C	L: 12–13.5 W: 6.5–8	Confirmed
*P. stuntzii*	Lageniform, fusoid-ampullaceous or fusiform-lanceolate with an elongated and flexuous neck. Figure 3E–F	Subellipsoid side view to subovoid face view Figure 3F	L: 6.6–9.1 W: 4.7–6.6 Figure 3F	L: 8–10.5 W: 5.5–7.5	Confirmed
*P. cyanescens*	Not pictured	Elongate-elliposoid Figure 3H	L: 10–10.6 W: 6.5–7.5 Figure 3H	L: 9–12 W: 5–8	Confirmed
*P. subaeruginosa*	Not pictured	Rhomboid to subrhombiod/subellipsoid Figure 3J	L: 10.5–11.9 W: 5.9–6.9 Figure 3J	L: 7.7–14 W: 6.6–8.5	Confirmed
*P. natalensis*	Not pictured	Broad ellipsoid on side and ovoid in face Figure 3L	L: 9–10 W: 5.5–6^d^ Figure 3L	L: 10–15 W: 7–9.4	Confirmed
*P. zapotecorum*	Not pictured	Oblong ellipsoid Figure 3O	L: 4.7–5.3^d^ W: 3.2–3.4^d^ Figure 3O	L: 5.5–8.8 W: 3.8–5.5	Confirmed

a Micromorphological features were characterized and described based on the vocabulary used in the literature (17, 18).

b L = Length.

c W = Width.

d Spore sizes fall outside the expected range for that species.

### Chemical Analysis by HPTLC

**Figure 4.**
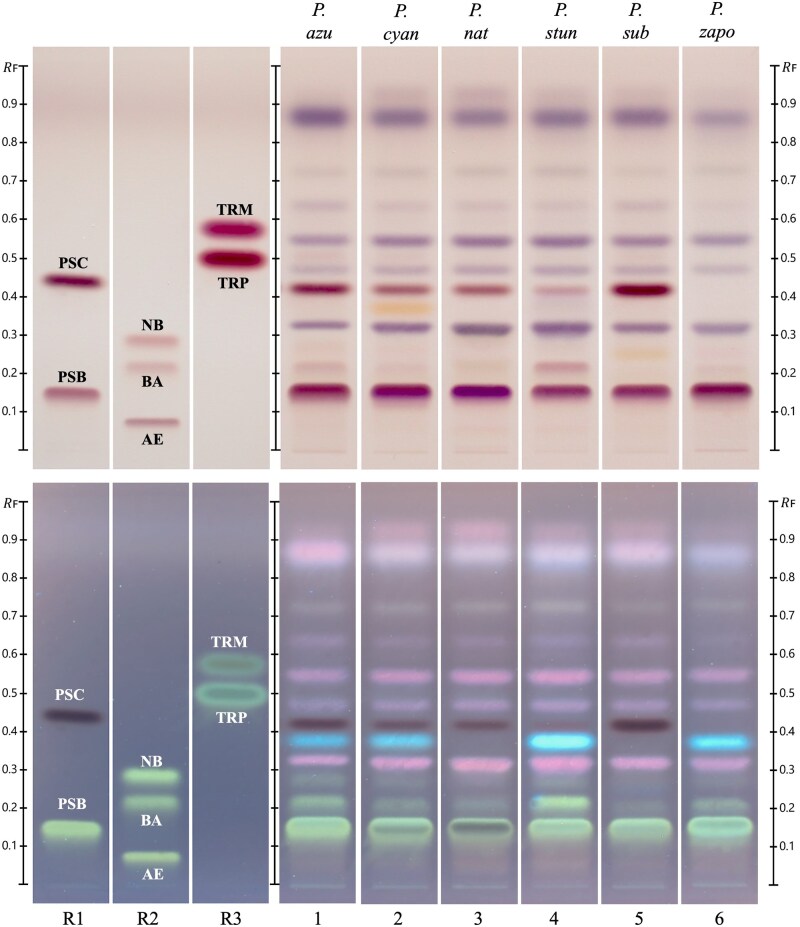
Mushroom HPTLC chemical profiles of six *Psilocybe* species. HPTLC chromatograms are visualized under white light (top) and under 366 nm light (bottom). R1, psilocin (R_F_ 0.45), psilocybin (R_F_ 0.15); R2, norbaeocystin (R_F_ 0.30), baeocystin (R_F_ 0.20), aeruginascin (R_F_ 0.06); R3, tryptamine (R_F_ 0.59), tryptophan (R_F_ 0.50); 1, *P. azurescens*; 2, *P. cyanescens;* 3, *P. natalensis*; 4, *P. stuntzii*; 5, *P. subaeruginosa*; 6, *P. zapotecorum*.

The HPTLC analysis identified key psychoactive compounds in mushrooms across the six successfully cultivated *Psilocybe* species (Figure 4). Distinct bands corresponding to reference standards were observed, confirming the presence of psilocybin, psilocin, baeocystin, and trace amounts of norbaeocystin and aeruginascin. Tryptamine and tryptophan, precursors to psilocybin, did not produce visible bands in any of the species (Figure 4). Figure 4 highlights notable interspecies differences under white light and 366 nm following derivatization. *P. subaeruginosa* exhibited a more pronounced psilocin band, suggesting higher concentrations of this compound in that species. A relatively strong band corresponding to psilocin was also observed in *P. azurescens,* followed closely by *P. cyanescens* and *P. natalensis,* with a less prominent band in *P. stuntzii,* and no visible band in *P. zapotecorum* (Figure 4). Baeocystin was detected in *P. stuntzii* and *P. azurescens,* with faint bands observed in *P. cyanescens, P. natalensis,* and *P. zapotecorum,* with a barely discernible band in *P. subaeruginosa.* Norbaeocystin presence was minor across all species, while aeruginascin was not visibly observed. All species displayed a pronounced band corresponding to psilocybin (Figure 4).

HPTLC profiles provide a qualitative visual representation of overall chemical diversity among species; however, this comparison is based on a single track per species. Quantification of psilocybin and psilocin in each species is depicted in Table 3, and the data reveals the following pattern: average psilocybin levels were highest in *P. zapotecorum,* followed by *P. azurescens, P. cyanescens, P. natalensis,* and *P. subaeruginosa* with the lowest levels in this dataset seen in *P. stuntzii*. Psilocin showed a different pattern, with the highest levels observed in *P. natalensis,* followed by *P. subaeruginosa, P. azurescens, P. cyanescens, P. zapotecorum,* and *P. stuntzii* (Table 3).

**Table 3.** Summary of psilocybin (PSB) and psilocin (PSC) % dry (w/w) contents for six *Psilocybe* species

Species	PSB High %	PSB Low %	PSB Mean % ± SD	PSC High %	PSC Low %	PSC Mean % ± SD	N (Samples)
*P. azurescens*	1.77	0.81	1.33 ± 0.325	0.29	0.08	0.20 ± 0.080	12
*P. cyanescens*	1.32	0.82	1.06 ± 0.181	0.29	0.04	0.12 ± 0.090	12
*P. natalensis*	1.34	0.62	1.03 ± 0.262	0.38	0.26	0.30 ± 0.045	5
*P. stuntzii*	1.11	0.45	0.84 ± 0.191	0.12	0.06	0.09 ± 0.026	15
*P. subaeruginosa*	1.58	0.67	1.01 ± 0.250	0.45	0.10	0.26 ± 0.097	14
*P. zapotecorum*	1.89	1.09	1.46 ± 0.213	0.17	0.02	0.10 ± 0.056	25

**Figure 5.**
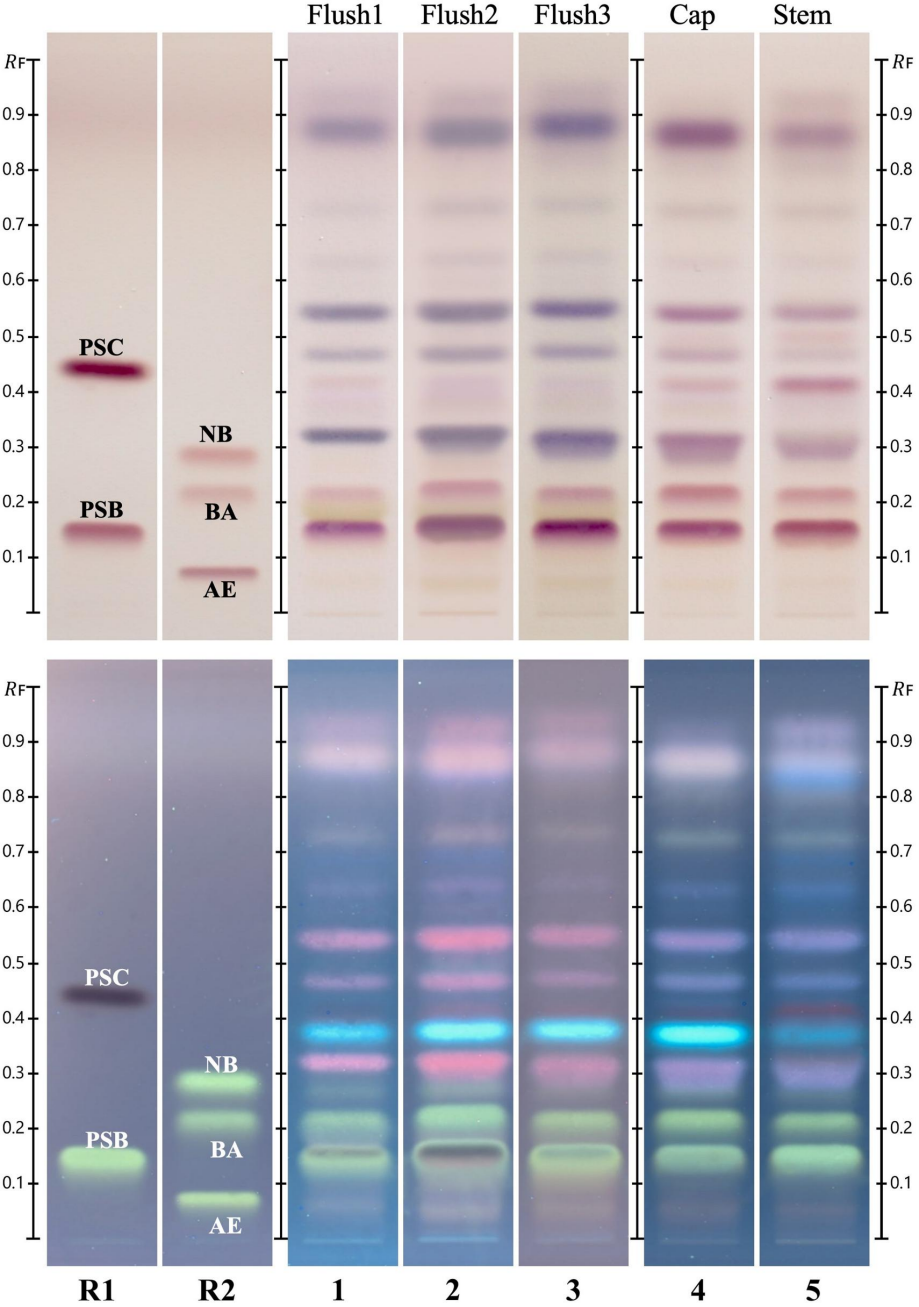
HPTLC chemical profiles of *Psilocybe stuntzii* mushrooms, from different flushes and comparison of the stem and cap. HPTLC chromatograms are visualized under white light (top) and under 366 nm light (bottom). R1, psilocin (R_F_ 0.45), psilocybin (R_F_ 0.15); R2, norbaeocystin (R_F_ 0.30), baeocystin (R_F_ 0.20), aeruginascin (R_F_ 0.06); 1, first flush; 2, second flush; 3, third flush; 4, *P. stuntzii* cap; 5, *P. stuntzii* stem.

Every crop or batch of cultivated mushrooms has a well-defined growing schedule. At some point in this schedule, mushrooms will emerge in flushes, separated by 7–21 days depending on the species. Typically, a single crop can produce two to three flushes before the substrate is exhausted. The stability of a *P. stuntzii* cultivar’s composition across three flushes was assessed through their HPTLC chromatograms, which demonstrated consistent chemical profiles with stable levels of psilocin, psilocybin (Figure 5). HPTLC chromatograms of *P. stuntzii* show distinct differences in compound abundances between the stem and cap (Figure 5). For example, the fluorescent band found at R_F_ 0.38 is more intense in the cap than in the stem. This strongly fluorescent substance (also observed under 366 nm light prior to derivatization) is currently being investigated as a putative ß-carboline, previously identified in certain *Psilocybe* species (11, 13, 22). Although this study does not report quantitative stem and cap data, preliminary HPTLC analysis indicates differences in psilocybin and psilocin content, as seen in a prior study on *P. cubensis* and an investigation on *P. zapotecorum* stems and caps (23, 24). These differences demonstrate the importance of considering mushroom morphology, specifically the stem-to-cap ratio, as well as developmental stages when analyzing compound concentrations. Variations in harvesting practices, such as leaving behind large portions of the stem, can influence the resulting compound profile. Consequently, studies that do not standardize or report these morphological characteristics may introduce large variability, thereby impacting the reproducibility and comparability of results.

**Figure 6.**
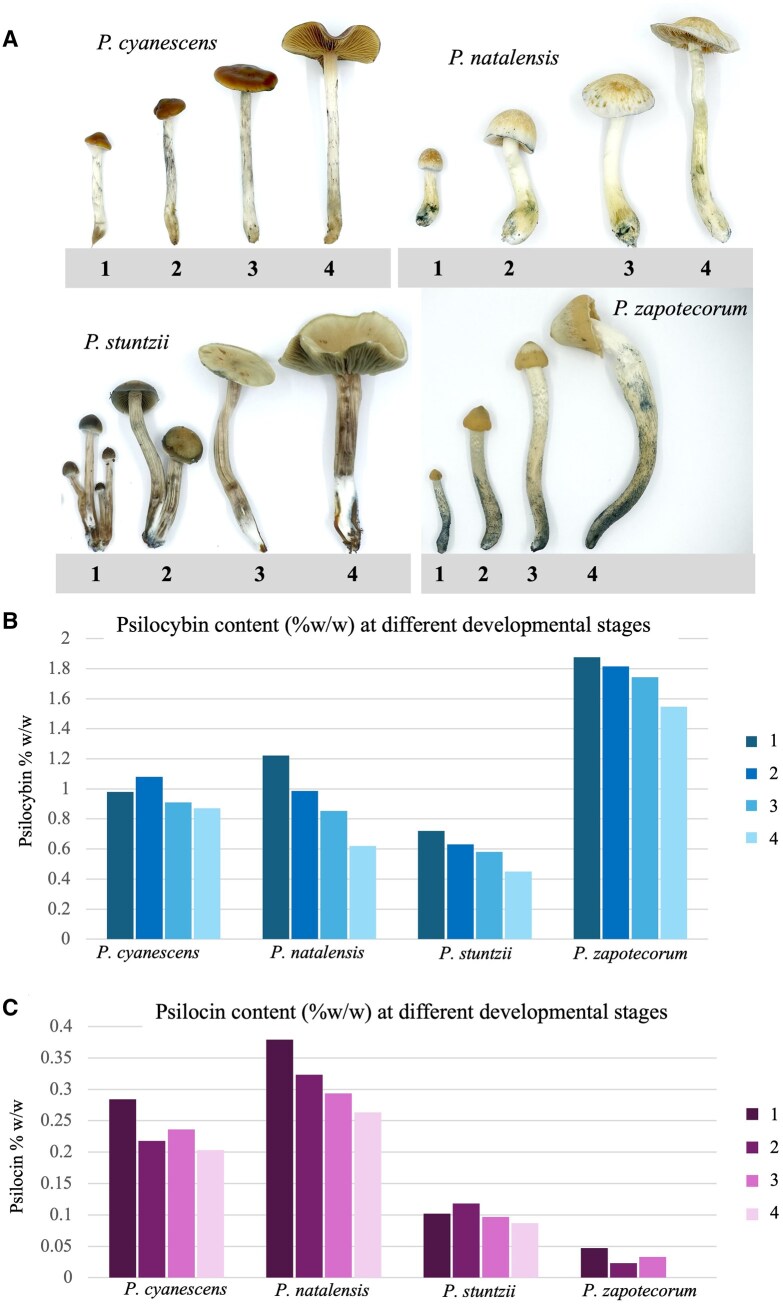
Psilocybin and psilocin content in four different *Psilocybe* species, at four stages of fruiting body development. Different developmental stages of *P. cyanescens, P. natalensis, P, stuntzii,* and *P. zapotecorum*, grown indoors under controlled conditions (A). Decreasing content of psilocybin (B) and psilocin (C), across different developmental stages in four *Psilocybe* species. Numbers in graphs (B and C) correspond to developmental stages portrayed in (A). Mushroom photos shown in (A) have slight differences in scale and thus may not accurately portray size differences between species.

The psilocybin and psilocin contents of mushrooms harvested at various developmental stages are shown in Figure 6. Each species of mushroom was harvested from a single substrate bag beginning with the pre-opening of the cap, with the partial veil still present in some of the species (Figure 6A-1). The next stage was an open cap in campanulate form (Figure 6A-2). In stage three the cap was convex to plane (Figure 6A-3), and in the fourth stage the cap was plane to uplifted (Figure 6A-4). Our analysis revealed that as *P. cyanescens*, *P. natalensis*, *P. stuntzii*, and *P. zapotecorum* mushrooms matured, there was a noticeable decrease in both psilocybin (Figure 6B) and psilocin (Figure 6C) concentrations. Although these findings are preliminary and based on limited data, they suggest that the timing of the harvest could influence the concentration of psychoactive compounds. This result would also indicate that assays of psychoactive compounds in wild and cultivated mushrooms should note the developmental stage to accurately present concentration data. Further investigations are necessary to determine the optimal harvest time to achieve consistent levels of these compounds, which is crucial for maintaining product potency.

### Freezer Storage and Psilocybin Degradation

**Figure 7.**
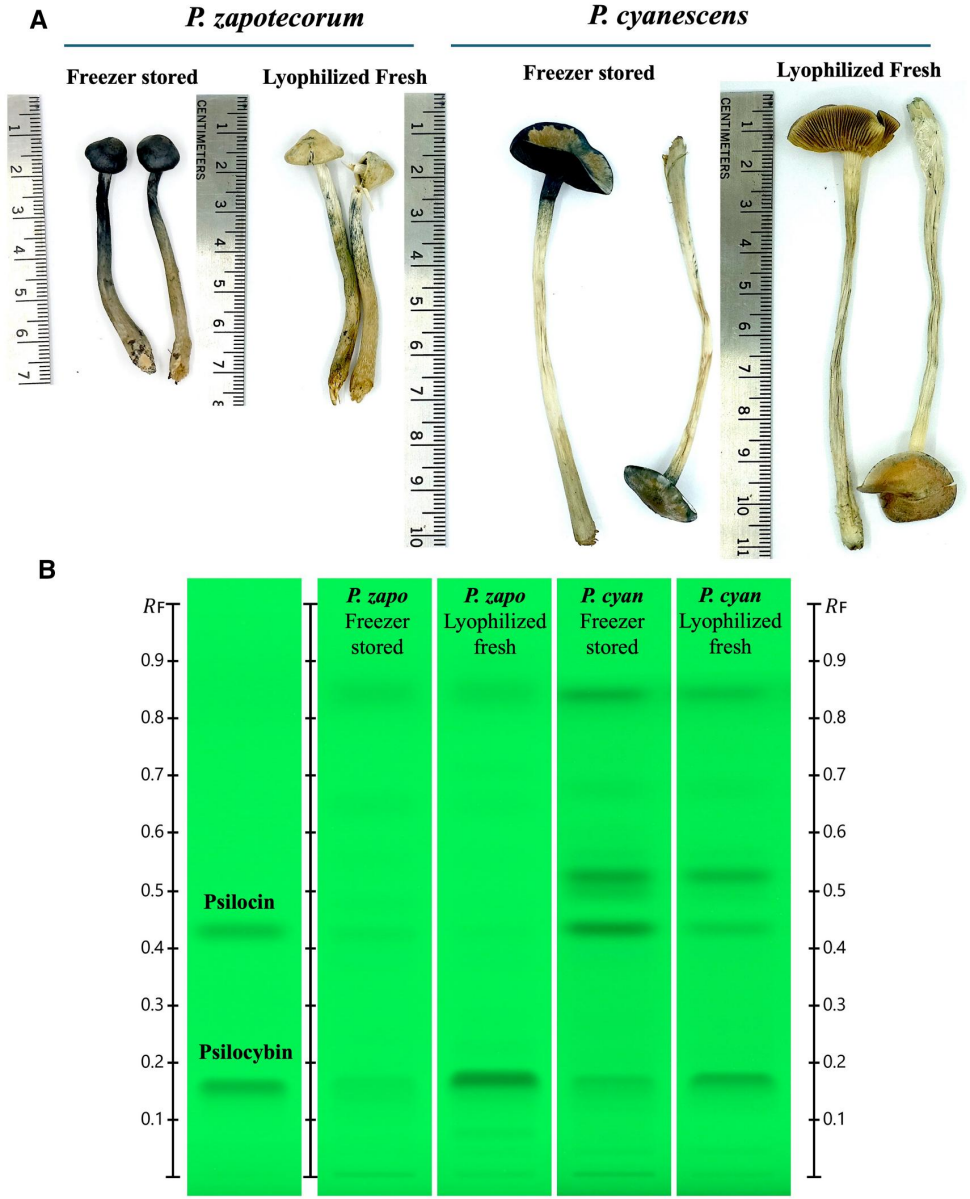
Psilocybin and psilocin content in mushrooms after storage at −20°C. Fresh-harvested *P. zapotecorum* and *P. cyanescens* lyophilized either immediately after harvest (lyophilized fresh) or after 24 h of storage in the freezer, at −20°C (freezer stored) (A). HPTLC 254 nm chromatogram depicting psilocybin and psilocin levels in freshly lyophilized and freezer-stored *P. zapotecorum* and *P. cyanescens* (B).

Mushrooms stored at −20°C for 24 h developed a deep blue color upon thawing (Figure 7A). This visual bluing does not necessarily correlate with psilocin levels, as some species displayed substantial increases in psilocin with only marginal bluing. Conversely, *P. zapotecorum* is shown in Figure 7 to become intensely blue upon thawing despite containing only marginal psilocin levels. Further experiments are necessary to quantify these effects with greater accuracy and establish the underlying mechanisms involved in color development. Freezing samples at −20°C for 24 h before freeze-drying led to substantial changes in psilocybin and psilocin content compared to samples lyophilized immediately after harvest (Figure 7B). In *P. zapotecorum*, the psilocybin content dropped from 1.29% in freshly lyophilized samples to 0.08% in freezer-stored samples. Conversely, psilocin levels increased from 0.04% in the fresh samples to 0.08% after freezer storage. For *P. cyanescens*, freezer storage reduced psilocybin from 1.16% to 0.27%, while psilocin content increased from 0.31% to 0.77%, which exceeded the calibration curve upper range of 0.5%.

## Conclusions

This study successfully demonstrates the cultivation of various *Psilocybe* mushroom species under controlled indoor conditions and provides a detailed chemical analysis using HPTLC. The species cultivated at the time of writing include *P. azurescens*, *P. cyanescens*, *P. natalensis*, *P. stuntzii*, *P. subaeruginosa*, and *P. zapotecorum*. Our efforts to cultivate more species are ongoing, and our recent exploration of different substrate preferences has led to a successful fruiting of *Psilocybe mexicana* and *Panaeolus cyanescens.* Both these species have yielded fruiting bodies in initial experiments and show promising cultivation traits. Other species have been more challenging to cultivate; for instance, the creation of two multispore cultures of *P. ovoideocystidiata* from different spore print sources produced strong vegetative mycelial growth in substrates and casing but have yet to produce a single mushroom. Thus, significant effort is still required for the optimization of substrate and growth conditions for some species, in addition to further collection of spore prints and tissue cultures which will improve our chances of developing stable and productive cultivars.

The HPTLC analysis identified key psychoactive compounds, including psilocybin, psilocin, norbaeocystin, baeocystin, and aeruginascin, across these species. Our findings highlight differences in the concentrations of these compounds among different *Psilocybe* species. The study also reveals the impact of developmental stages on the concentrations of psilocybin and psilocin, with a general trend of decreasing levels as the mushrooms mature. This information is crucial for optimizing harvest times to ensure consistent concentrations of desired compounds.

The detailed chemical profiles generated through HPTLC can guide the development of genetically stable cultivars and can inform commercial cultivation practices for producing *Psilocybe* mushrooms with consistent chemical profiles. By monitoring the stability of compounds across multiple flushes, as demonstrated with *P. stuntzii*, we can develop cultivation practices that produce reliable, high-quality psilocybin mushroom products and enable growers to obtain several flushes from a single substrate, thus maximizing productivity. The freezer storage of fresh harvested mushrooms was shown to have a major effect on psilocybin stability, and this practice should be avoided. The differences in compound distribution between stems and caps highlight the need for comprehensive profiling of different mushroom parts across different species. Reports on active compounds should consider accounting for the mass contributions from stems and caps, as differences in mushroom parts can significantly influence the analytical results, especially in studies with small sample sizes.

This research provides a foundation for the standardized cultivation and chemical profiling of *Psilocybe* species, contributing valuable insights into their chemical diversity. Future work will expand on these findings by incorporating additional analytical techniques, such as HPLC and mass spectrometry, to provide a more comprehensive profile of psychoactive compounds like ß-carbolines. The goal is to develop genetically stable *Psilocybe* cultivars that can guarantee consistent productivity and comprehensive chemical profiles optimized for both research and health practitioner purposes.

## CRediT Author Statement

Coleton Windsor (Conceptualization [Equal], Data curation [Lead], Formal analysis [Lead], Investigation [Equal], Methodology [Lead], Validation [Lead], Visualization [Lead]), Anna Evgenya Kreynes (Visualization [Equal]), Jeff S. Chilton (Conceptualization [Lead], Data curation [Lead], Formal analysis [Lead], Funding acquisition [Lead], Investigation [Lead], Methodology [Lead], Project administration [Lead], Resources [Lead], Supervision [Lead]), Bill A. Chioffi (Conceptualization [Lead], Funding acquisition [Lead], Project administration [Lead], Resources [Lead], Supervision [Lead]), and Christopher Niebergall (Investigation [Equal], Methodology [Equal], Project administration [Equal], Resources [Equal], Validation [Equal], Visualization [Equal]), Kelsey Dodds (Data curation [Equal], Formal analysis [Equal], Investigation [Supporting], Validation [Supporting], Visualization [Equal])

## Conflict of Interest

All authors are employed by Nammex. Nammex has a financial interest in the development of *Psilocybe* cultivars for future research. The authors declare no other conflicts of interest.
