# Correction to: Development of *Psilocybe* Mushroom Species Reference Material—Cultivation Parameters and Chemical Profiles

**DOI:** 10.1093/jaoacint/qsaf104

**Published:** 2025-12-22

This is a correction to: Coleton Windsor, Anna E Kreynes, Jeff S Chilton, William A Chioffi, Christopher Niebergall, Kelsey Dodds, Development of *Psilocybe* Mushroom Species Reference Material—Cultivation Parameters and Chemical Profiles, *Journal of AOAC INTERNATIONAL*, 2025; https://doi.org/10.1093/jaoacint/qsaf007

The following changes have been made to the originally published manuscript.

Under heading **Chemical Analysis**, section c) the mobile phase ratio was incorrectly reported as 1-butanol, acetic acid, and H_2_O in a ratio of “6:1:1 (v/v/v)”. The correct composition is 1-butanol, acetic acid, and H_2_O in a ratio of “6.5:1.75:1.75 (v/v/v)”. This correction does not affect results, figures or conclusions of the article.

